# Impact of intravenous fluid composition on outcomes in patients with systemic inflammatory response syndrome

**DOI:** 10.1186/s13054-015-1045-z

**Published:** 2015-09-12

**Authors:** Andrew D. Shaw, Carol R. Schermer, Dileep N. Lobo, Sibyl H. Munson, Victor Khangulov, David K Hayashida, John A. Kellum

**Affiliations:** Dept of Anesthesiology, Vanderbilt University Medical Center, 1215 21st Avenue S., Suite 5160 MCE NT, Office 5163, Campus Box 8274, Nashville, TN 37232-8274 USA; Former Employee Baxter Healthcare Corporation, 1 Baxter Parkway, Deerfield, IL 60015 USA; Gastrointestinal Surgery, National Institute for Health Research Nottingham Digestive Diseases Biomedical Research Unit, Nottingham University Hospitals and University of Nottingham, Queen’s Medical Centre, Derby Road, Nottingham, NG7 2UH UK; Boston Strategic Partners, Inc., 4 Wellington Street, Boston, MA 02118 USA; Center for Critical Care Nephrology, Department of Critical Care Medicine, University of Pittsburgh, 3550 Terrace Street, Pittsburgh, PA 15261 USA

## Abstract

**Introduction:**

Intravenous (IV) fluids may be associated with complications not often attributed to fluid type. Fluids with high chloride concentrations such as 0.9 % saline have been associated with adverse outcomes in surgery and critical care. Understanding the association between fluid type and outcomes in general hospitalized patients may inform selection of fluid type in clinical practice. We sought to determine if the type of IV fluid administered to patients with systemic inflammatory response syndrome (SIRS) is associated with outcome.

**Methods:**

This was a propensity-matched cohort study in hospitalized patients receiving at least 500 mL IV crystalloid within 48 hours of SIRS. Patient data was extracted from a large multi-hospital electronic health record database between January 1, 2009, and March 31, 2013. The primary outcome was in-hospital mortality. Secondary outcomes included length of stay, readmission, and complications measured by ICD-9 coding and clinical definitions. Outcomes were adjusted for illness severity using the Acute Physiology Score. Of the 91,069 patients meeting inclusion criteria, 89,363 (98 %) received 0.9 % saline whereas 1706 (2 %) received a calcium-free balanced solution as the primary fluid.

**Results:**

There were 3116 well-matched patients, 1558 in each cohort. In comparison with the calcium-free balanced cohort, the saline cohort experienced greater in-hospital mortality (3.27 % vs. 1.03 %, *P* <0.001), length of stay (4.87 vs. 4.38 days, *P* = 0.016), frequency of readmission at 60 (13.54 vs. 10.91, *P* = 0.025) and 90 days (16.56 vs. 12.58, *P* = 0.002) and frequency of cardiac, infectious, and coagulopathy complications (all *P* <0.002). Outcomes were defined by administrative coding and clinically were internally consistent. Patients in the saline cohort received more chloride and had electrolyte abnormalities requiring replacement more frequently (*P* <0.001). No differences were found in acute renal failure.

**Conclusions:**

In this large electronic health record, the predominant use of 0.9 % saline in patients with SIRS was associated with significantly greater morbidity and mortality compared with predominant use of balanced fluids. The signal is consistent with that reported previously in perioperative and critical care patients. Given the large population of hospitalized patients receiving IV fluids, these differences may confer treatment implications and warrant corroboration via large clinical trials.

**Trial registration:**

NCT02083198 clinicaltrials.gov; March 5, 2014

**Electronic supplementary material:**

The online version of this article (doi:10.1186/s13054-015-1045-z) contains supplementary material, which is available to authorized users.

## Introduction

The concentration of chloride in 0.9 % saline is supraphysiological [1] and infusion of moderate to large volumes has been shown to produce hyperchloremic acidosis [2–12]. Hyperchloremic acidosis has been associated with adverse physiological effects in both animals and human volunteers [2, 9, 10, 13, 14]. However, small human trials have failed to show detrimental clinical outcomes when it has been compared with balanced crystalloids [15–18].

In animals, experimental hyperchloremic acidosis induces shock, is proinflammatory, and reduces survival in sepsis [19–22]. Recent studies have suggested that both hyperchloremia [23] and infusion of chloride-rich fluids [24, 25] have detrimental effects on clinical outcomes in postoperative and critically ill patients. A propensity-matched study of surgical patients who received 0.9 % saline or a calcium-free balanced crystalloid on the day of surgery showed a higher proportion of 0.9 % saline recipients developing complications [24]. A similar analysis comparing balanced crystalloids with 0.9 % saline in patients with sepsis found that balanced crystalloids were associated with improved survival [26]. A study evaluating the impact of restricting high-chloride fluids in intensive care unit (ICU) patients showed that chloride restriction led to a lower incidence of acute kidney injury and need for renal replacement therapy, but showed no differences in hospital mortality or length of stay [27].

Although there is a growing body of evidence to suggest that 0.9 % saline may adversely impact outcomes in critically ill and surgical patients, studies evaluating the impact of high-chloride fluids in hospitalized patients with lower illness severity are lacking. The purpose of this study was to determine if the hazards associated with 0.9 % saline that appear to occur in surgical and ICU patients also occur in a broader patient population receiving intravenous (IV) fluid therapy. The aim of the study was to determine whether, among patients with systemic inflammatory response syndrome (SIRS), use of 0.9 % saline as an early fluid choice was associated with adverse outcomes, when compared with a regimen composed of more physiologic IV solutions.

## Methods

### Overview

We examined a large US electronic health record (EHR) database (HealthFacts®, Cerner Corp., Kansas City, MO, USA) to identify hospitalized patients who met at least two SIRS criteria and received at least 500 mL of IV fluid within 48 hours of first developing SIRS. We included patients aged ≥18 years with a length of stay of at least 24 hours. We defined SIRS as the presence of tachycardia [heart rate (HR) >90 bpm] along with at least one of the following on the same day: (1) temperature >38 °C or <36 °C, (2) respiratory rate ≥20 breaths/minute or PaCO_2_ ≤32 mmHg, or (3) leukocytes ≥12,000 or ≤4,000 cells/mm^3^. This modified SIRS definition was chosen because HR was the most populated field of the SIRS criteria and allowed us to initially select patients from a dataset of >1 million subjects. Two propensity-matched cohorts were created based upon the type of isotonic crystalloid received. The study protocol and analysis plan were approved (prior to data extraction) by the Duke University institutional review board with waiver of requirement for written informed consent, and was registered on the clinicaltrials.gov website (NCT02083198).

### Inclusion criteria and cohorts

**Fig. 1:**
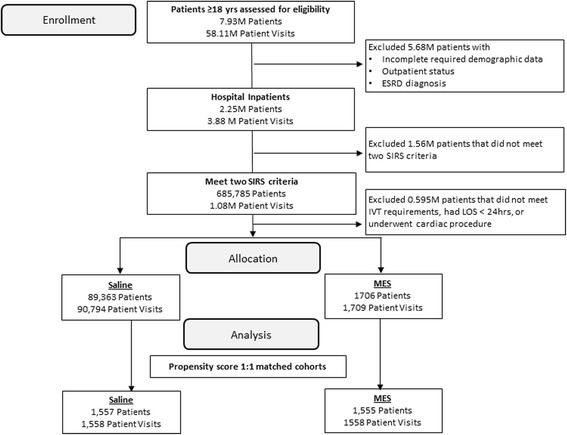
Cohort selection

Included patients were required to receive ≥500 mL of the cohort-qualifying crystalloid solution within 2 days of meeting SIRS criteria. In order to determine if the associations with fluid type were similar to findings in the surgical population, patients selected for the balanced fluid cohort must have received ≥500 mL of a calcium-free balanced crystalloid (Plasma-Lyte® [Baxter Healthcare, Deerfield, IL, USA] or Normosol® [Hospira, Lake Forest, IL, USA]). Patients selected for the saline cohort must have received ≥500 mL 0.9 % saline and must not have received any calcium-free balanced fluid (Fig. 1). To capture the full picture of crystalloid administered, patients in either cohort were also allowed to have received additional balanced fluids or saline in the 72 hours following SIRS criterion with the exception that the saline cohort could not receive calcium-free balanced fluid. These other fluid types and volumes for non-dextrose-containing IV fluid (calcium-free balanced, 0.9 % saline, lactated Ringer’s, and 0.45 % saline) were summed over a 3-day time period post SIRS qualification. IV fluid orders for bag volumes ≤250 mL were not included in the total volumes because they are frequently used for drug admixture.

### Exclusion criteria

Patients receiving >1 L of IV fluid the day prior to SIRS qualification were excluded. Also excluded were those who had cardiac surgery (part of another study), a diagnosis of end-stage renal disease, or receipt of any colloid or hypertonic saline.

### Data source

HealthFacts*®* is a de-identified EHR database that is an aggregate of administrative data [International Classification of Diseases, Ninth Revision (ICD-9)] and clinical/quantitative data (medication and diagnostic test orders and laboratory results). A subset of inpatients from January 1, 2009, through March 31, 2013, was utilized.

### Data processing

Analysis inclusion required the presence of patient identifier, age, race, and gender, admission and discharge date/time, discharge status and SIRS qualification, and IV fluid data. Diagnoses and procedures were captured if present, as well as microbiology tests if positive. Laboratory results were available and the absence of a laboratory value was assumed normal. For multiple readings, the most abnormal reading was utilized.

### Outcomes

Outcomes were evaluated by administratively coded data and corroborated by clinically defined parameters. Mortality was the primary outcome of interest. Secondary outcomes, chosen based on animal models [13, 14, 19–22], small human trials [2–5, 9, 10, 15–18] and physiological argument, included in-hospital complications, electrolyte and acid–base disturbances, length of stay, and readmission. Administrative outcomes were defined by ICD-9 diagnosis codes and categorized as: (1) cardiac, (2) hemorrhage, (3) infection, (4) gastrointestinal (GI), (5) neurologic, (6) acute renal failure, (7) respiratory failure, and (8) new organ failure (a composite of new organ system failure). Outcomes were determined by codes not denoted as “present on admission” (Table S1 in Additional file 1).

Clinical outcomes were operationally defined and grouped as follows (Table S2 in Additional file 2). Cardiac complications: (1) dysrhythmia – infusion of IV antiarrhythmics on day of SIRS qualification through 3 days, (2) cardiac stress – abnormal troponin, (3) congestive heart failure (CHF) – order for ECHO or brain natriuretic peptide (BNP) >600 pg/mL, and same day diuretic use, (4) cardiac failure – vasopressor use within 3 days of SIRS qualification, and (5) cardiac Sequential Organ Failure Assessment (SOFA) score [28]. Hemorrhage complications: (1) hemoglobin decrease of >20 g/L/24 hours, (2) transfusion by ICD-9 procedure codes, (3) coagulopathy by abnormal prothrombin time-international normalized ratio (PT-INR) in the absence of warfarin, thrombocytopenia of <150,000 × 10^9^/l, or abnormal D-dimer, (4) SOFA coagulation score. Infection was defined by leukocyte count >12,000 × 10^9^ cells/l within 1 day of date of culture and administration of antibiotics within 3 days of culture. Positive cultures were specific to each infection: (1) pneumonia-tracheal aspirate or bronchoalveolar lavage, (2) bacteremia/sepsis by blood culture, and (3) urinary tract infection by urine culture. Respiratory failure was assessed using SOFA pulmonary scores [28]. GI complications were defined by: (1) acute cholecystitis, and (2) SOFA liver score. Acute kidney injury was defined by Kidney Disease Improving Global Outcomes (KDIGO) criteria using serum creatinine only [29]. The baseline creatinine used was the lowest creatinine in the 7 days prior to the SIRS qualifying event.

Electrolytes monitored included magnesium, potassium, sodium, and ionized calcium. Electrolytes were designated as abnormal when outside of the following ranges: (1) Mg <0.70 mmol/L; >1.0 mmol/L, (2) K <3.5 mmol/L; >5.0 mmol/L, (3) Na <136 mmol/L; >145 mmol/L, (4) ionized Ca <1.1 mmol/L; >1.4 mmol/L. Patients with low magnesium, potassium, or ionized calcium who received IV magnesium, IV calcium, or IV or oral potassium were counted as having received replacement. Acidosis was defined operationally and measured in three ways: (1) hyperchloremic acidosis: arterial blood pH <7.35 and serum chloride >110 mmol/L, (2) metabolic acidosis as pH <7.35 and bicarbonate <22 mmol/L, (3) lactic acidosis as venous or arterial lactate >2.0 mmol/L.

### Comorbidities and severity of illness

Comorbidities were defined using ICD-9 diagnostic codes and collated using the Elixhauser algorithm [30]. To ensure mutual exclusivity of the comorbidities and outcomes, the renal failure unspecified (ICD-9 code 586) comorbidity was removed because it overlapped with the administrative outcome. Peripheral vascular disease was modified to remove ICD-9 code 557.9 due to overlap with a GI administrative outcome. The electrolyte abnormalities comorbidity was not included because it was a measured outcome.

To further evaluate severity of illness, the Acute Physiology Score (APS) from the Acute Physiology and Chronic Health Evaluation (APACHE) II score was calculated for each patient [31] using data collected on the day of SIRS qualification and 1 day prior, using the higher of the two. Where clinical data were missing for an APS variable, it was assumed to be normal.

### Controlling for bias and confounding: propensity scores, cohort matching and adjusted outcomes models

To account for potential selection bias, we developed a propensity score [32] representing the probability that a patient would receive balanced crystalloid based on patient and hospital characteristics and comorbidities. The score was calculated using logistic regression with backward inclusion of the covariates. The patients in the saline and balanced cohorts were then matched 1:1 using a case–control greedy matching algorithm [33].

All administrative and clinical outcomes were compared between the matched cohorts. Continuous characteristics were compared by Student’s *t* test and categorical parameters were compared by χ^2^ test. A *P* value of less than 0.05 was considered statistically significant. To account for the impact of severity of acute illness, the APS was used to adjust all outcomes. In addition, outcome models were also adjusted for variables that were different (*P* <0.1) between cohorts after propensity matching. Outcome models are reported as odds ratios (ORs) with 95 % confidence intervals (CIs). Data were analyzed using SAS/Base software, Version 9.3 (SAS Institute Inc., Cary, NC, USA).

## Results

Propensity scoring was able to match 1558 of 1709 patients receiving balanced fluid to patients receiving saline (Table 1). The hospital characteristics that remained significantly different after the match were admission source, payor, and census region. For patient characteristics, the frequency for CHF was greater in the saline cohort, and hypertension greater in the balanced cohort. Post-match APS scores remained significantly different, hence the above hospital and patient characteristics that remained different at a *P* <0.1 and APS were used to adjust the outcome models.

**Table 1:** Matched baseline hospital and patient characteristics for balanced and saline cohorts

Patient demographics	Saline	Balanced	*P* value (two-sided)
	n = 1558	n = 1558	
Age group^a^			0.43
18–35	16.88 (263)	17.97 (280)	
36–50	19.06 (297)	19.45 (303)	
51–64	25.8 (402)	27.15 (423)	
65–80	27.54 (429)	26.44 (412)	
80+	10.72 (167)	8.99 (140)	
Gender			0.13
Male	44.09 (687)	41.46 (646)	
Female	55.91 (871)	58.54 (912)	
Race			0.56
Black	11.62 (181)	10.46 (163)	
Other	14.44 (225)	14.25 (222)	
White	73.94 (1152)	75.29 (1173)	
Bed size			0.06
0–199	9.88 (154)	9.18 (143)	
200–399	39.99 (623)	44.22 (689)	
400+	50.13 (781)	46.6 (726)	
Admission source			<0.001
Emergency	1.03 (16)	0.71 (11)	
Healthcare facility	5.07 (79)	7.83 (122)	
Non-healthcare facility	71.69 (1117)	74.01 (1153)	
Other/Unknown	22.21 (346)	17.46 (272)	
Admission type			0.09
Elective	52.37 (816)	56.1 (874)	
Emergency	47.37 (738)	43.77 (682)	
Other/Unknown	0.26 (4)	0.13 (2)	
Payor			0.03
Commercial	13.22 (206)	16.56 (258)	
Medicare/Medicaid	37.48 (584)	37.03 (577)	
Other	49.29 (768)	46.41 (723)	
Urban			0.09
Yes	98.27 (1531)	98.97 (1542)	
No	1.73 (27)	1.03 (16)	
Teaching			0.09
Yes	85.43 (1331)	87.48 (1363)	
No	14.57 (227)	12.52 (195)	
Census region			0.002
Northeast	65.53 (1021)	59.82 (932)	
South	10.85 (169)	11.3 (176)	
West	23.62 (368)	28.88 (450)	
Patients with surgical procedures^b^	15.53 (242)	14.96 (233)	0.65
Congestive heart failure	3.85 (60)	2.37 (37)	0.02
Valvular heart disease	1.93 (30)	1.22 (19)	0.11
Pulmonary circulation disease	0.26 (4)	0.26 (4)	1.00
Peripheral vascular disease	2.82 (44)	2.57 (40)	0.67
Paralysis	0.9 (14)	0.77 (12)	0.69
Other neurological disorders	3.27 (51)	3.15 (49)	0.84
Chronic pulmonary disease	7.96 (124)	9.11 (142)	0.25
Diabetes w/o chronic complications	7 (109)	7.96 (124)	0.31
Diabetes w/chronic complications	1.86 (29)	1.28 (20)	0.20
Hypothyroidism	3.15 (49)	3.98 (62)	0.21
Renal failure	2.95 (46)	2.25 (35)	0.22
Liver disease	0.96 (15)	1.48 (23)	0.19
Acquired immune deficiency syndrome	0.19 (3)	0.19 (3)	1.00
Lymphoma	1.03 (16)	0.39 (6)	0.03
Metastatic cancer	0.64 (10)	0.77 (12)	0.67
Solid tumor w/o metastasis	1.73 (27)	1.8 (28)	0.89
Rheumatoid arthritis/collagen vas	2.7 (42)	1.73 (27)	0.07
Coagulopathy	1.09 (17)	1.35 (21)	0.51
Obesity	7.12 (111)	8.66 (135)	0.11
Weight loss	1.93 (30)	1.41 (22)	0.26
Chronic blood loss anemia	1.28 (20)	1.35 (21)	0.88
Deficiency anemias	4.24 (66)	5.01 (78)	0.31
Alcohol abuse	1.93 (30)	2.12 (33)	0.70
Drug abuse	1.6 (25)	1.35 (21)	0.55
Psychoses	1.22 (19)	1.48 (23)	0.53
Depression	7.64 (119)	7.25 (113)	0.68
Hypertension	17.2 (268)	22.08 (344)	<0.001

^a^Age used as a continuous variable in the propensity score model

^b^Surgical procedure not used within the propensity score model

The median amount of fluid received over 72 hours after SIRS qualification was similar in both cohorts (Table 2). Patients in both groups received a combination of 0.45 % saline, lactated Ringer’s, and 0.9 % saline. Patients in the calcium-free balanced cohort received a significantly lower mean chloride load (112 mmol/L vs. 147 mmol/L), reflecting the chloride content of their early predominant IV fluid.

**Table 2:** Seventy-two-hour fluid volumes and chloride load by matched cohort

Matched 1:1 Calcium-free balanced: 0.9 % saline cohort fluids					
	Calcium-free balanced^a^	0.45 % Saline	Lactated Ringer’s	0.9 % Saline	Totals^b^
Calcium-free balanced cohort					
+ n (%)	1558 (100 %)	73 (4.7 %)	548 (35.2 %)	857 (55.6 %)	1558 (100 %)
Average mmol/L	178.4	88.4	172.8	232.3	112.4
Median mmol/L	98	77 .0	109	154	109.2
Average vol. (median)	1.8 L (1.0 L)	1.1 L (1.0 L)	1.6 L (1.0 L)	1.5 L (1.0 L)	3.3 L (2.0 L)
Vol. range	0.5–10.4 L	0.4–3.0 L	0.5–13.5 L	0.5–16.0 L	0.5–19.8 L
0.9 % Saline cohort					
n (%)	Exclusion criteria	54 (3.5 %)	348 (22.3 %)	1558 (100 %)	1558 (100 %)
Average mmol/L		89.3	219	246.5	147.4
Median mmol/L		77	174.4	154	154.0
Average vol. (median)		1.2 L (1.0 L)	2.0 L (1.6 L)	1.6 L (1.0 L)	2.1 L (2 L)
Vol. range		0.5–4.0 L	0.3–10.3 L	0.5–11.6 L	0.5–12.0 L

^+^Note n represents number of patients in the cohort receiving the particular fluid i.e., 73 patients in the calcium-free cohort received 0.45 % saline

^a^Calcium-free balanced crystalloid (e.g., Plasma-Lyte or Normosol: Na 140 mmol/L, Cl 98 mmol/L, K 5.0 mmol/L, Mg 1.5 mmol/L, gluconate 23 mmol/L, acetate 27 mmol/L)

^b^Monitored fluids (calcium-free balanced, 0.45 % saline, lactated Ringer’s, 0.9 % saline)

### Unadjusted outcomes

Table 3 shows the administrative outcomes demonstrating that calcium-free balanced fluid use was associated with a lower rate of major complications. Specifically, patients receiving calcium-free balanced fluid experienced fewer cardiac, respiratory, infectious, and new organ failure complications. Among the significant outcome differences, the most frequent cardiac complication was atrial fibrillation, the most frequent respiratory complication was acute respiratory failure, the most frequent infectious complication was pneumonia followed by sepsis, and the most frequent new organ failure was acute renal failure, followed by congestive heart failure. Although acute renal failure contributed to the composite of new organ failure, by itself it was not significantly lower in the calcium-free balanced cohort. In addition, no differences were found in administrative outcomes for hemorrhage, GI, or neurological complications.

**Table 3:** Administrative and clinical outcomes by fluid cohort

Outcome/Complication	Saline	Balanced	Unadjusted odds ratio (95 % confidence interval)	Adjusted odds ratio (95 % confidence interval)
	(n = 1558) % (n)	(n = 1558) % (n)		
Administrative outcomes				
Cardiac	8.66 (135)	4.43 (69)	0.488 (0.362–0.659)	0.51 (0.378–0.689)
Hemorrhage	0.71 (11)	0.51 (8)	0.726 (0.291–1.809)	0.781 (0.311–1.961)
Infectious	10.14 (158)	6.03 (94)	0.569 (0.436–0.742)	0.618 (0.471–0.809)
Gastrointestinal	5.46 (85)	5.13 (80)	0.938 (0.685–1.284)	0.969 (0.707–1.328)
Neurologic	1.16 (18)	0.83 (13)	0.72 (0.352–1.474)	0.817 (0.395–1.69)
Acute renal failure	4.36 (68)	3.34 (52)	0.757 (0.524–1.093)	0.917 (0.625–1.344)
Respiratory failure	5.46 (85)	3.47 (54)	0.622 (0.439–0.882)	0.692 (0.485–0.986)
New organ failure	9.11 (142)	7.12 (111)	0.765 (0.59–0.991)	0.872 (0.667–1.141)
Clinical outcomes				
Hospital mortality	3.27 (51)	1.03 (16)	0.307 (0.174–0.54)	0.378 (0.211–0.676)
30-day readmissions	9.5 (148)	7.64 (119)	0.788 (0.612–1.014)	0.802 (0.622–1.032)
60-day readmissions	13.54 (211)	10.91 (170)	0.782 (0.63–0.97)	0.798 (0.643–0.991)
90-day readmissions	16.56 (258)	12.58 (196)	0.725 (0.593–0.886)	0.741 (0.606–0.907)
Cardiac				
Dysrhythmia	10.65 (166)	6.87 (107)	0.618 (0.48–0.797)	0.649 (0.502–0.838)
Cardiac stress	6.42 (100)	2.05 (32)	0.306 (0.204–0.458)	0.341 (0.226–0.515)
Heart failure	4.04 (63)	1.48 (23)	0.356 (0.219–0.576)	0.401 (0.246–0.653)
Hemorrhage/Hematologic				
Coagulopathy^a^	11.09 (150)	7.71 (106)	0.67 (0.516–0.87)	0.717 (0.55–0.934)
Received blood transfusion	2.92 (46)	2.27 (36)	0.777 (0.5–1.209)	0.851 (0.544–1.33)
Bleeding	20.09 (313)	19.96 (311)	0.992 (0.832–1.182)	1.074 (0.897–1.286)
Infectious				
Pneumonia	6.03 (94)	2.12 (33)	0.337 (0.225–0.504)	0.393 (0.259–0.596)
Sepsis	10.53 (164)	5.65 (88)	0.509 (0.389–0.666)	0.568 (0.431–0.75)
Urinary tract infection	5.91 (92)	4.69 (73)	0.783 (0.571–1.074)	0.904 (0.653–1.252)
Line infection	0.77 (12)	0	-	-
Gastrointestinal				
Cholecystitis	4.75 (74)	3.53 (55)	0.734 (0.514–1.048)	0.836 (0.58–1.203)
Renal				
Acute kidney injury	5.46 (85)	4.43 (69)	0.803 (0.580–1.112)	0.920 (0.658–1.286)
Electrolyte abnormalities				
Low magnesium with replacement	4.36 (68)	2.12 (33)	0.474 (0.311–0.723)	0.546 (0.355–0.84)
High magnesium	4.36 (68)	3.4 (53)	0.772 (0.535–1.113)	0.95 (0.647–1.393)
Low potassium with replacement	14.12 (220)	7.51 (117)	0.494 (0.39–0.625)	0.551 (0.429–0.706)
High potassium	9.44 (147)	6.87 (107)	0.708 (0.546–0.918)	0.813 (0.621–1.066)
Low sodium	39.41 (614)	30.62 (477)	0.678 (0.585–0.787)	0.707 (0.608–0.822)
High sodium	7 (109)	6.03 (94)	0.854 (0.642–1.135)	1.004 (0.746–1.351)
Low calcium with replacement	1.28 (20)	0.96 (15)	0.748 (0.381–1.466)	1.052 (0.517–2.14)
Metabolic acidosis				
Lactic (pH and lactate)	0.71 (11)	0.26 (4)	0.362 (0.115–1.139)	0.528 (0.161–1.728)
Metabolic (pH and bicarbonate)	2.76 (43)	1.22 (19)	0.435 (0.252–0.75)	0.617 (0.342–1.115)
Hyperchloremic (pH and chloride)	3.47 (54)	1.22 (19)	0.344 (0.203–0.583)	0.449 (0.256–0.786)

^a^Excluded patients receiving warfarin

Table 3 also shows the clinical outcomes. Calcium-free balanced fluid use was associated with lower mortality, shorter hospital stay, lower 60- and 90-day readmission rates, and a lower rate of major complications. The calcium-free balanced group had significantly fewer cardiac complications as measured by dysrhythmias, cardiac stress, and congestive heart failure (BNP and diuretic use definition). There were fewer patients with infectious complications of pneumonia, sepsis, and line infections. SOFA scoring showed that cardiac, liver, and hematologic failure were significantly lower in balanced patients (Table S3 in Additional file 3). Although there was a difference in coagulopathy favoring calcium-free balanced fluid, there was no difference in hemoglobin decrease. Acute kidney injury was not different between groups by KDIGO stage. Length of hospital stay was 0.48 days shorter in the calcium-free balanced group (4.38 vs. 4.86 days, *P* = 0.016). Readmission data showed a 30-day readmission rate of 7.6 % in the balanced cohort and 9.5 % in the saline cohort (*P* = 0.06). The 60- and 90-day readmission rates were significantly lower in the balanced cohort vs. saline (10.9 % vs. 13.5 % (*P* = 0.025) and 12.6 % vs. 16.6 % (*P* = 0.002) at 60 and 90 days, respectively).

The frequency of electrolyte abnormalities was lower for balanced patients. Moreover, the percentages of patients with a low serum magnesium and low serum potassium receiving replacement were lower in the balanced fluid cohort (*P* <0.001). In addition, the frequency of hyperkalemia was lower (*P* = 0.012) in the balanced fluid cohort.

The different measures of acidosis showed that hyperchloremic acidosis and metabolic acidosis (as defined by low pH and low bicarbonate) were more frequent in the saline cohort (both *P* <0.001). Although the percentage of patients with lactic acidosis was not different between cohorts (0.71 % saline vs. 0.26 % balanced; *P* = 0.07), more patients receiving saline had lactate levels ordered (5.3 % saline vs. 1.4 % balanced, *P* <0.001).

### Model adjustment

**Fig. 2:**
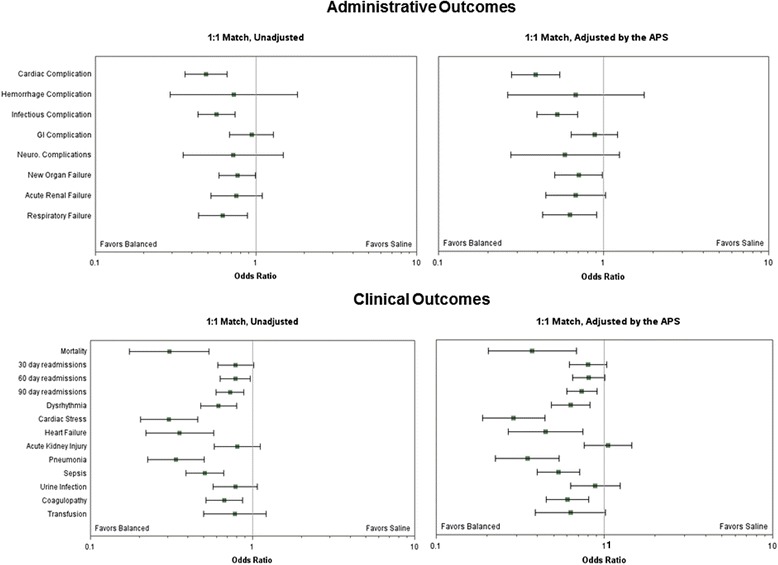
Administrative and clinical outcomes unadjusted and adjusted for Acute Physiology Score

Figure 2 shows the administrative and clinical outcomes unadjusted and adjusted for APS and variables with a *P* <0.10 post-match. APS differed between groups post-match (4.77 saline vs. 4.37 calcium-free balanced, *P* <0.001). The only administrative outcome that became nonsignificant when adjusted for APS and incompletely matched variables was 60-day readmission. Adjustment of the clinical outcomes affected hyperkalemia and hypocalcemia, requiring replacement and metabolic acidosis, such that they were no longer significant. No administrative or clinical outcome adjusted for APS significantly favored saline.

## Discussion

This observational study provides further support of a possible signal of harm associated with 0.9 % saline, which may be avoided by the use of more physiological fluids. This study evaluated a broader population than our study of surgical patients and corroborated many of the findings [24]. It appears that the use of saline in patients with SIRS is associated with increased mortality, increased cardiac, infectious, and coagulopathy complications, and an increased need for electrolyte replacement. The inclusion of outcomes measured using clinically defined parameters in this study further strengthens these findings and validates our prior analyses of administrative datasets [24]. Importantly, none of the administrative or clinical outcomes favored the use of 0.9 % saline.

As opposed to our study of major abdominal surgery [24] where we evaluated patients who received exclusively saline or calcium-free balanced fluid, here, although we selected the cohorts based on receipt of calcium-free balanced fluid or saline early in relation to the SIRS event, we intentionally included all non-dextrose-containing crystalloids to make our analysis more generalizable with clinical practice, wherein clinicians use many different types of fluids. Both cohorts received a combination of fluids, but the saline patients did not receive calcium-free balanced fluids. Although many patients in both cohorts received a mix of such that just over one-half of patients in the balanced cohort received saline and roughly 22–35 % in both groups received Ringer’s lactate, the initial fluid choice of balanced versus unbalanced resulted in a substantial difference in the ultimate chloride load delivered. We speculate the important exposure difference is likely delivered chloride load, a concept which supports our findings from the larger dataset of 98,000 patients that chloride and volume contribute independently to mortality [25].

In this study, we failed to observe a difference in renal failure/acute kidney injury by ICD-9 coding or KDIGO scoring. We interpret the lack of corroboration of other studies showing increased risk for acute kidney injury or renal failure with saline as these patients may have been at overall lower baseline risk than the major surgery and critically ill patients, and hence may have been better able to deal with the hazard. The frequency of elevated creatinine was low (3–4 %) and hence there may be no effect of fluid type on renal risk at the volumes administered in this study. Indeed, in healthy rats, exposure to saline had no effect on kidney function, whereas in animals with sepsis the same volumes resulted in worsening of acute kidney injury [22].

Two criticisms of our prior study [24] were that patients in the saline cohort received more blood and more fluid, which may have driven the worse outcomes. However, in the current study, there was no difference in the frequency of blood transfusion, and the median fluid volumes were similar, but complication rates still favored balanced fluid. Our findings support Raghunathan’s study of patients with sepsis, which showed that as the proportion of saline increased, mortality increased, and this association appeared to be independent of volume [26]. In the present study, the calcium-free balanced cohort received a substantially lower chloride load, lending support for the concept that mortality and complications are related to fluid composition and not solely to the fluid volume received per se.

Access to clinical data allowed us to provide face validity to the concept that hyperchloremic acidosis was also associated with a higher rate of complications. Our findings support McCluskey’s recent study showing an association of hyperchloremia with increased mortality [23]. These investigators reported mortality rates of 3.0 % in their hyperchloremic group and 1.9 % in their normochloremic group [23], quite similar to our findings of 3.3 % mortality in the saline cohort versus 1.1 % in the calcium-free balanced cohort.

The coagulation abnormalities seen in the SOFA score and by laboratory measures are consistent with abnormalities seen in trauma patients receiving saline [34] and with lower transfusion rates found in surgical patients receiving physiological fluids [17, 24]. However, the clinical implications of these abnormalities are unclear since transfusion did not differ between cohorts.

We believe an important implication of these data regards the exposed population. Intravenous fluid therapy is one of the most common inpatient interventions prescribed, and 0.9 % saline is the most common fluid used around the world [35, 36]. Thus, if there is a hazard associated with 0.9 % saline, then it could affect large numbers of patients. Given the absence of evidence supporting the use of 0.9 % saline, we believe its use should be limited to the few indications where it is likely of value (e.g., hypovolemic hypochloremic alkalosis).

Observational analyses are inevitably limited by bias and confounding. We attempted to minimize confounding by using a robust propensity-matching algorithm based on potential confounders at the hospital and patient level. For the administrative outcomes, we ensured that outcomes did not overlap with comorbidity codes. The array of patient and hospital information available should have minimized residual effects from unmeasured confounders. Moreover, severity of illness and inadequately matched potential confounders were used to adjust the outcomes. Confounding by indication should have been minimized by our timing of treatment related to the SIRS episode.

The clinical outcomes could be considered more robust than the administrative outcomes, but they are not perfect. The use of the electronic health record did not allow us to obtain results of items such as electrocardiograms (ECGs) or imaging findings in order to fully inform clinical definitions, but we do consider the use of laboratory data and medications to be robust support corroborating the administrative data.

## Conclusions

This evaluation of patients with SIRS receiving crystalloids of different composition adds to the growing body of real-world data showing that 0.9 % saline may not be innocuous when used for volume replacement. These observational studies have generated sufficient questions to warrant a large-scale clinical trial. The recent UK National Institute for Health and Care Excellence guidelines on intravenous fluid therapy [37] recognized the potential hazards of large-volume infusions of 0.9 % saline, and in the absence of large-scale randomized trials, it recommended further research in the area. Although this study does not elucidate the mechanisms leading to the findings, it contributes to the body of research evaluating the potential hazards of 0.9 % saline and its mitigation via the use of commonly available alternative solutions.

## Key messages

- Fluids with high chloride concentrations such as 0.9 % saline have been associated with adverse outcomes in surgery and critical care. Understanding the association between fluid type and outcomes in less ill hospitalized patients may inform selection of fluid type in clinical practice.
- In a large electronic health record, the early use of 0.9 % saline in patients with SIRS was associated with significantly greater morbidity and mortality compared with predominant use of balanced fluids.

## Additional files

Additional file 1: Table S1. Summary of administrative outcomes ICD-9 diagnosis and procedure code definitions. This table summarizes the administrative outcomes by ICD-9 diagnosis and procedure codes, grouped by category. (DOCX 26 kb) Additional file 2: Table S2. Summary of clinical outcomes definitions and criteria. This table summarizes and defines clinical outcomes, grouped by category. (DOCX 18 kb) Additional file 3: Table S3. SOFA outcomes by treatment group. This table shows the prevalence of SOFA outcomes, categorized by treatment group. (DOCX 12 kb)

## Abbreviations

APS: Acute Physiology Score
BNP: brain natriuretic peptide
CHF: congestive heart failure
EHR: electronic health record
GI: gastrointestinal
ICD-9: International Classification of Diseases, Ninth Revision
ICU: intensive care unit
IV: intravenous
SIRS: systemic inflammatory response syndrome
SOFA: Sequential Organ Failure Assessment
