# Erratum to: Impact of intravenous fluid composition on outcomes in patients with systemic inflammatory response syndrome

**DOI:** 10.1186/s13054-016-1187-7

**Published:** 2016-01-20

**Authors:** Andrew D. Shaw, Carol R. Schermer, Dileep N. Lobo, Sibyl H. Munson, Victor Khangulov, David K Hayashida, John A. Kellum

**Affiliations:** 1 Department of Anesthesiology, Vanderbilt University Medical Center, 1215 21st Avenue S., Suite 5160 MCE NT, Office 5163, Campus Box 8274, Nashville, 37232-8274 TN USA; 2 Former Employee Baxter Healthcare Corporation, 1 Baxter Parkway, Deerfield, 60015 IL USA; 3 Gastrointestinal Surgery, National Institute for Health Research Nottingham Digestive Diseases Biomedical Research Unit, Nottingham University Hospitals and University of Nottingham, Queen’s Medical Centre, Derby Road, Nottingham, NG7 2UH UK; 4 Boston Strategic Partners, Inc., 4 Wellington Street, Boston, 02118 MA USA; 5 Center for Critical Care Nephrology, Department of Critical Care Medicine, University of Pittsburgh, 3550 Terrace Street, Pittsburgh, 15261 PA USA

After the publication of this article it has been brought to our attention that the data in Table 3 (Table 1 in this Erratum) and in the Additional file 3: Table S3 (Additional File 1 in this Erratum) contained mistakes as detailed below. These mistakes have been corrected in this Erratum.

**Table 3:** Administrative and clinical outcomes by fluid cohort

Outcome/Complication	Saline (*n* = 1558) % (n)	Balanced (*n* = 1558) % (n)	Unadjusted odds ratio (95 % CI)	Adjusted odds ratio (95 % CI)
Administrative Outcomes				
Cardiac	8.66 (135)	4.43 (69)	0.488 (0.362–0.659)	0.387 (0.276–0.544)
Hemorrhage	0.71 (11)	0.51 (8)	0.726 (0.291–1.809)	0.681 (0.264–1.757)
Infectious	10.14 (158)	6.03 (94)	0.569 (0.436–0.742)	0.526 (0.396–0.699)
Gastrointestinal	5.46 (85)	5.13 (80)	0.938 (0.685–1.284)	0.881 (0.636–1.220)
Neurologic	1.16 (18)	0.83 (13)	0.72 (0.352–1.474)	0.585 (0.275–1.245)
Acute renal failure	4.36 (68)	3.34 (52)	0.757 (0.524–1.093)	0.676 (0.447–1.023)
Respiratory failure	5.46 (85)	3.47 (54)	0.622 (0.439–0.882)	0.624 (0.429–0.908)
New organ failure	9.11 (142)	7.12 (111)	0.765 (0.59–0.991)	0.707 (0.506–0.986)
Clinical Outcomes				
Hospital mortality	3.27 (51)	1.03 (16)	0.307 (0.174–0.54)	0.373 (0.204–0.681)
30–day readmissions	9.5 (148)	7.64 (119)	0.788 (0.612–1.014)	0.801 (0.618–1.036)
60–day readmissions	13.54 (211)	10.91 (170)	0.782 (0.63–0.97)	0.804 (0.645–1.004)
90–day readmissions	16.56 (258)	12.58 (196)	0.725 (0.593–0.886)	0.735 (0.598–0.904)
Cardiac				
Dysrhythmia	10.65 (166)	6.87 (107)	0.618 (0.48–0.797)	0.632 (0.485–0.823)
Cardiac stress	6.42 (100)	2.05 (32)	0.306 (0.204–0.458)	0.289 (0.188–0.483)
Heart failure	4.04 (63)	1.48 (23)	0.356 (0.219–0.576)	0.447 (0.268–0.747)
Hemorrhage/Hematologic				
Coagulopathy^a^	11.09 (150)	7.71 (106)	0.67 (0.516–0.87)	0.602 (0.450–0.806)
Received blood transfusion	2.92 (46)	2.27 (36)	0.777 (0.5–1.209)	0.629 (0.389–1.018)
Bleeding	20.09 (313)	19.96 (311)	0.992 (0.832–1.182)	1.134 (0.942–1.365)
Infectious				
Pneumonia	6.03 (94)	2.12 (33)	0.337 (0.225–0.504)	0.349 (0.226–0.539)
Sepsis	10.53 (164)	5.65 (88)	0.509 (0.389–0.666)	0.534 (0.401–0.711)
Urinary tract infection	5.91 (92)	4.69 (73)	0.783 (0.571–1.074)	0.884 (0.632–1.237)
Line infection	0.77 (12)	0	–	–
Gastrointestinal				
Cholecystitis	4.75 (74)	3.53 (55)	0.734 (0.514–1.048)	0.753 (0.517–1.098)
Renal				
Acute Kidney Injury	5.46 (85)	4.43 (69)	0.803 (0.580–1.112)	1.052 (0.760–1.455)
Electrolyte Abnormalities				
Low magnesium with replacement	4.36 (68)	2.12 (33)	0.474 (0.311–0.723)	0.565 (0.362–0.880)
High magnesium	4.43 (69)	3.4 (53)	0.760 (0.528–1.095)	0.822 (0.553–1.221)
Low potassium with replacement	14.12 (220)	7.51 (117)	0.494 (0.39–0.625)	0.510 (0.393–0.660)
High potassium	9.44 (147)	6.87 (107)	0.708 (0.546–0.918)	0.848 (0.643–1.118)
Low sodium	39.41 (614)	30.62 (477)	0.678 (0.585–0.787)	0.703 (0.602–0.821)
High sodium	7 (109)	6.03 (94)	0.854 (0.642–1.135)	0.965 (0.710–1.311)
Low calcium with replacement	1.16 (18)	0.77 (12)	0.664 (0.319–1.383)	1.125 (0.534–2.369)
Metabolic Acidosis				
Lactic (pH and lactate)	0.71 (11)	0.26 (4)	0.362 (0.115–1.139)	0.397 (0.106–1.487)
Metabolic (pH and bicarbonate)	2.76 (43)	1.22 (19)	0.435 (0.252–0.75)	0.749 (0.399–1.403)
Hyperchloremic (pH and chloride)	3.47 (54)	1.22 (19)	0.344 (0.203–0.583)	0.477 (0.262–0.868)

^a^Excluded patients receiving warfarin

The following values have been corrected in Table 3 (Table 1 in this Erratum):

- the entire column (far right) "Adjusted Odds Ratio (95 % CI)"
- under Electrolyte Abnormalities, the value for "High Mg" within the column "Saline n (%)" and the values for "Unadjusted Odds Raio (95 % CI)" and "Adjusted Odds Ratio (95 % CI)"
- under Electrolyte Abnormalities, ALL values for the row labelled "Low Ca with replacement"

The single value for SOFA CV Level 2 "Saline cohort n (%)" in Table S3 (Additional File 1 in this Erratum) has been corrected.

In addition, we noticed that the legend for Fig. 2 (Fig. 1 in this Erratum) was incorrectly given as “Administrative and clinical outcomes unadjusted and adjusted for Acute Physiology Score”. The correct legend for Fig. 2 is “Administrative and clinical outcomes unadjusted and adjusted for APS, Elixhauser comorbidities of congestive heart failure, hypertension and lymphoma, admission source, census region, and payer.” The labels above the right-hand side plots within the figure also needed corrections and have been updated in this erratum.

**Fig. 2:**
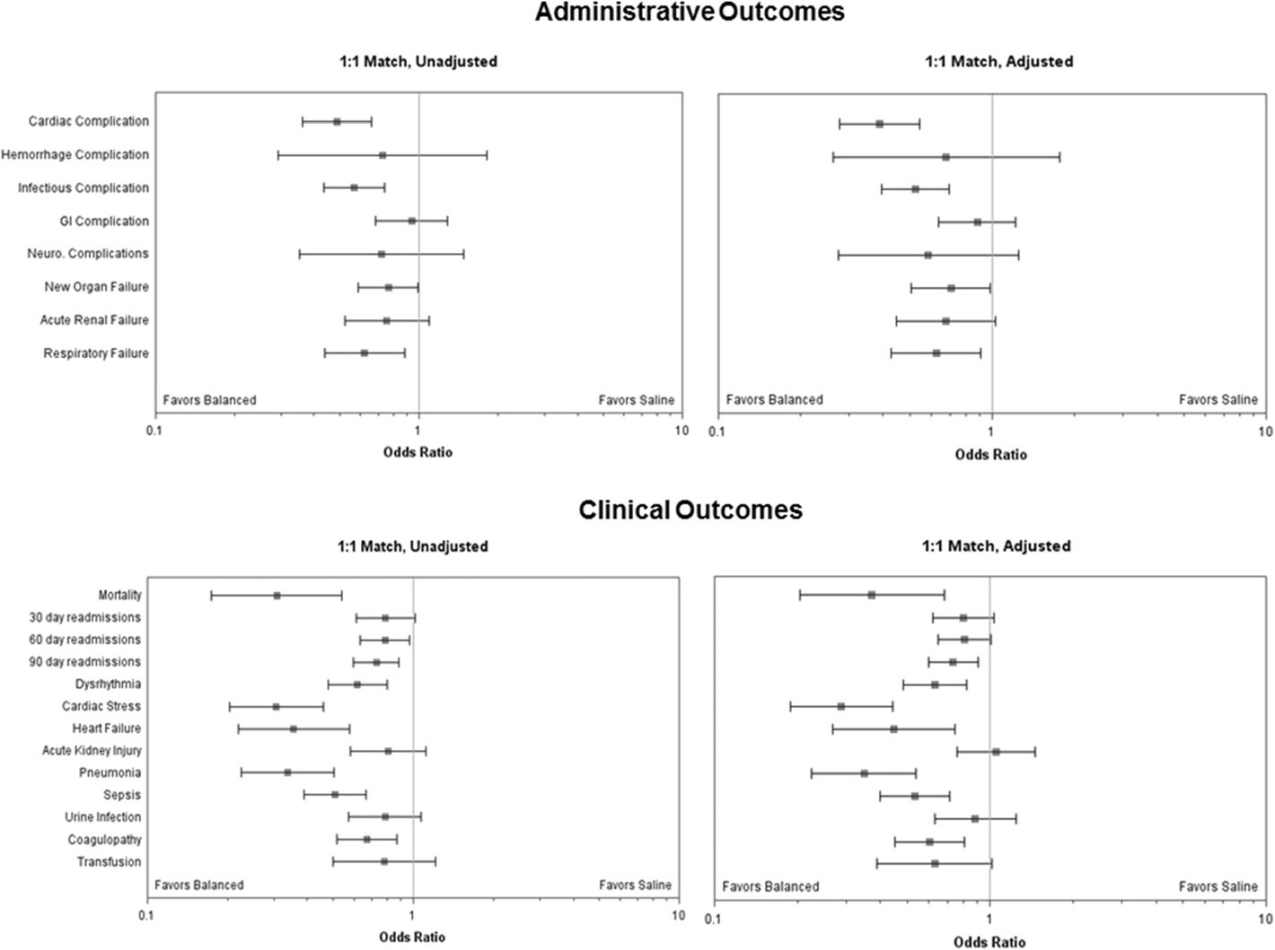
legend, edit to: Administrative and clinical outcomes unadjusted and adjusted for APS, Elixhauser comorbidities of congestive heart failure, hypertension and lymphoma, admission source, census region, and payer

## Additional file

Additional file 3: Table S3. SOFA Outcomes by Treatment Group. (DOCX 12 kb)
