# Supplementary material for: Impact of intravenous fluid composition on outcomes in patients with systemic inflammatory response syndrome
Source: Crit Care. 2015 Sep 12;19(1):334. doi: 10.1186/s13054-015-1045-z (PMC4570151; doi:10.1186/s13054-015-1045-z)
Supplement: Additional file 1: Table S1. — Summary of administrative outcomes ICD-9 diagnosis and procedure code definitions. This table summarizes the administrative outcomes by ICD-9 diagnosis and procedure codes, grouped by category. (DOCX 26 kb) [file 13054_2015_1045_MOESM1_ESM.docx]

| **Table S1.** Summary of administrative outcomes ICD-9 diagnosis and procedure code definitions | | | |
| --- | --- | --- | --- |
| **Administrative Complication** | **ICD-9 Code** | **ICD-9 Code Description** | **ICD-9 Code Type** |
| **Respiratory Failure** | 96.7x | Other continuous invasive mechanical ventilation | Procedure |
|  | 518.0 | Pulmonary collapse | Diagnosis |
|  | 518.1 | Interstitial emphysema | Diagnosis |
|  | 518.2 | Compensatory emphysema | Diagnosis |
|  | 518.3 | Pulmonary eosinophilia | Diagnosis |
|  | 518.4 | Acute edema of lung, unspecified | Diagnosis |
|  | 518.5 | Pulmonary insufficiency following trauma and surgery | Diagnosis |
|  | 518.51 | Acute respiratory failure following trauma and surgery | Diagnosis |
|  | 518.52 | Other pulmonary insufficiency, not elsewhere classified, following trauma and surgery | Diagnosis |
|  | 518.53 | Acute and chronic respiratory failure following trauma and surgery | Diagnosis |
|  | 518.6 | Allergic bronchopulmonary aspergillosis | Diagnosis |
|  | 518.7 | Transfusion related acute lung injury (TRALI) | Diagnosis |
|  | 518.8 | Other diseases of lung | Diagnosis |
|  | 518.81 | Acute respiratory failure | Diagnosis |
|  | 518.82 | Other pulmonary insufficiency, not elsewhere classified | Diagnosis |
|  | 518.83 | Chronic respiratory failure | Diagnosis |
|  | 518.84 | Acute and chronic respiratory failure | Diagnosis |
|  | 518.89 | Other diseases of lung, not elsewhere classified | Diagnosis |
|  | 31.1 | Temporary tracheostomy | Procedure |
| **Cardiac Complication** | 37.62 | Insertion of non-implantable heart assist system | Procedure |
|  | 37.66 | Insertion of implantable heart assist system | Procedure |
|  | 37.68 | Insertion of percutaneous external heart assist device | Procedure |
|  | 97.44 | Nonoperative removal of heart assist system | Procedure |
|  | 427 | Paroxysmal supraventricular tachycardia | Diagnosis |
|  | 427.1 | Paroxysmal ventricular tachycardia | Diagnosis |
|  | 427.3x | Atrial fibrillation and flutter | Diagnosis |
|  | 427.4x | Ventricular fibrillation and flutter | Diagnosis |
|  | 37.21 | Right heart cardiac catheterization | Procedure |
|  | 37.22 | Left heart cardiac catheterization | Procedure |
|  | 37.23 | Combined right and left heart cardiac catheterization | Procedure |
|  | 37.94 | ICD insertion | Procedure |
|  | 99.61 | Atrial cardioversion | Procedure |
|  | 99.62 | Other electric countershock of heart | Procedure |
|  | 410 | Acute myocardial infarction | Diagnosis |
|  | 410.0 | Acute myocardial infarction, of anterolateral wall | Diagnosis |
|  | 410.00 | Acute myocardial infarction, of anterolateral wall, episode of care unspecified | Diagnosis |
|  | 410.01 | Acute myocardial infarction, of anterolateral wall, initial episode of care | Diagnosis |
|  | 410.1 | Acute myocardial infarction, of other anterior wall | Diagnosis |
|  | 410.10 | Acute myocardial infarction, of other anterior wall, episode of care unspecified | Diagnosis |
|  | 410.11 | Acute myocardial infarction, of other anterior wall, initial episode of care | Diagnosis |
|  | 410.2 | Acute myocardial infarction, of inferolateral wall | Diagnosis |
|  | 410.20 | Acute myocardial infarction, of inferolateral wall, episode of care unspecified | Diagnosis |
|  | 410.21 | Acute myocardial infarction, of inferolateral wall, initial episode of care | Diagnosis |
|  | 410.3 | Acute myocardial infarction, of inferoposterior wall | Diagnosis |
|  | 410.30 | Acute myocardial infarction, of inferoposterior wall, episode of care unspecified | Diagnosis |
|  | 410.31 | Acute myocardial infarction, of inferoposterior wall, initial episode of care | Diagnosis |
|  | 410.4 | Acute myocardial infarction, of other inferior wall | Diagnosis |
|  | 410.40 | Acute myocardial infarction, of other inferior wall, episode of care unspecified | Diagnosis |
|  | 410.41 | Acute myocardial infarction, of other inferior wall, initial episode of care | Diagnosis |
|  | 410.5 | Acute myocardial infarction, of other lateral wall | Diagnosis |
|  | 410.50 | Acute myocardial infarction, of other lateral wall, episode of care unspecified | Diagnosis |
|  | 410.51 | Acute myocardial infarction, of other lateral wall, initial episode of care | Diagnosis |
|  | 410.6 | Acute myocardial infarction, true posterior wall infarction | Diagnosis |
|  | 410.60 | True posterior wall infarction, episode of care unspecified | Diagnosis |
|  | 410.61 | True posterior wall infarction, initial episode of care | Diagnosis |
|  | 410.7 | Acute myocardial infarction, subendocardial infarction | Diagnosis |
|  | 410.70 | Subendocardial infarction, episode of care unspecified | Diagnosis |
|  | 410.71 | Subendocardial infarction, initial episode of care | Diagnosis |
|  | 410.8 | Acute myocardial infarction, of other specified sites | Diagnosis |
|  | 410.80 | Acute myocardial infarction of other specified sites, episode of care unspecified | Diagnosis |
|  | 410.81 | Acute myocardial infarction of other specified sites, initial episode of care | Diagnosis |
|  | 410.9 | Acute myocardial infarction, unspecified site | Diagnosis |
|  | 410.90 | Acute myocardial infarction of unspecified site, episode of care unspecified | Diagnosis |
|  | 410.91 | Acute myocardial infarction of unspecified site, initial episode of care | Diagnosis |
| **Gastrointestinal Complication** | 574.0x | Calculus of gallbladder with acute cholecystitis | Diagnosis |
|  | 574.3x | Calculus of bile duct with acute cholecystitis | Diagnosis |
|  | 575 | Acute cholecystitis | Diagnosis |
|  | 557 | Acute vascular insufficiency of intestine | Diagnosis |
|  | 557.9 | Unspecified vascular insufficiency of intestine | Diagnosis |
|  | 578.xx | GI bleed | Diagnosis |
|  | 531.0x | Acute gastric ulcer with hemorrhage | Diagnosis |
|  | 531.2x | Acute gastric ulcer with hemorrhage and perforation | Diagnosis |
|  | 532.0x | Acute duodenal ulcer with hemorrhage | Diagnosis |
|  | 532.2x | Acute duodenal ulcer with hemorrhage and perforation | Diagnosis |
|  | 533.0x | Acute peptic ulcer with hemorrhage | Diagnosis |
|  | 533.2x | Acute peptic ulcer with hemorrhage and perforation | Diagnosis |
|  | 530.12 | Acute esophagitis | Diagnosis |
|  | 535.xx | Gastritis and duodentitis (**Excluding** codes 535.3 – Alcoholic Gastritis,  535.31 - Alcoholic Gastritis, without Mention of Hemorrhage, and  535.32 - Alcoholic Gastritis, with Hemorrhage) | Diagnosis |
|  | 9 | Infectious colitis, enteritis, and gastroenteritis | Diagnosis |
|  | 9.1 | Colitis, enteritis, and gastroenteritis of presumed infectious origin | Diagnosis |
|  | 536.2 | Persistent vomiting | Diagnosis |
|  | 564.3 | Vomiting following gastrointestinal surgery | Diagnosis |
|  | 787 | Nausea and vomiting | Diagnosis |
|  | 787.01 | Nausea with vomiting | Diagnosis |
|  | 787.03 | Vomiting alone | Diagnosis |
|  | 787.02 | Nausea alone | Diagnosis |
| **Hemorrhage** | 998.1 | Hemorrhage or hematoma complicating a procedure | Diagnosis |
|  | 998.11 | Hemorrhage complicating a procedure | Diagnosis |
|  | 998.12 | Hematoma complicating a procedure | Diagnosis |
|  | 287.9 | Unspecified hemorrhagic conditions | Diagnosis |
|  | 459.0 | Hemorrhage, unspecified | Diagnosis |
|  | 958.2 | Secondary and recurrent hemorrhage | Diagnosis |
| **Neurologic Complication** | 431 | Intracerebral hemorrhage | Diagnosis |
|  | 434.1x | Cerebral embolism | Diagnosis |
|  | 434.9x | Cerebral artery occlusion, unspecified | Diagnosis |
|  | 997.02 | Iatrogenic cerebrovascular infarction or hemorrhage, postoperative stroke | Diagnosis |
|  | 292.81 | Drug-induced delirium | Diagnosis |
|  | 293.0 | Delirium due to conditions classified elsewhere | Diagnosis |
|  | 293.1 | Subacute delirium | Diagnosis |
| **Renal Failure** | 584.xx | Acute renal failure | Diagnosis |
|  | 586 | Renal failure, unspecified | Diagnosis |
|  | 588.8 | Other specified disorders resulting from impaired renal function | Diagnosis |
|  | 588.9 | Unspecified disorder resulting from impaired renal function | Diagnosis |
|  | 39.95 | Dialysis | Procedure |
|  | 38.95 | Venous catheter for renal dialysis | Procedure |
| **Infectious Complication** | 038.xx | Septicemia | Diagnosis |
|  | 790.7 | Bacteremia | Diagnosis |
|  | 041.xx | Bacterial infections unspecified site | Diagnosis |
|  | 997.31 | Ventilator associated pneumonia | Diagnosis |
|  | 481 | Pneumococcal pneumonia [Streptococcus pneumoniae pneumonia] | Diagnosis |
|  | 482.41 | Methicillin susceptible pneumonia due to Staphylococcus aureus | Diagnosis |
|  | 482.8x | Pneumonia due to other specified bacteria | Diagnosis |
|  | 484.xx | Pneumonia in infectious diseases classified elsewhere | Diagnosis |
|  | 486 | Pneumonia, organism unspecified | Diagnosis |
|  | 510.xx | Empyema | Diagnosis |
|  | 567.22 | Peritoneal abscess | Diagnosis |
|  | 567.29 | Other suppurative peritonitis | Diagnosis |
|  | 569.5x | Abscess of intestine | Diagnosis |
|  | 519.2 | Mediastinitis | Diagnosis |
|  | 995.91 | Sepsis | Diagnosis |
|  | 995.92 | Severe sepsis | Diagnosis |
|  | 998.3x | Disruption of wound/dehiscence | Diagnosis |
|  | 999.31 | Other and unspecified infection due to central venous catheter | Diagnosis |
|  | 999.32 | Bloodstream infection due to central venous catheter | Diagnosis |
|  | 999.33 | Local infection due to central venous catheter | Diagnosis |
| **New Organ Failure** | 402.01 | Malignant hypertensive heart disease with heart failure | Diagnosis |
|  | 402.11 | Benign hypertensive heart disease with heart failure | Diagnosis |
|  | 402.91 | Unspecified hypertensive heart disease with heart failure | Diagnosis |
|  | 428 | Congestive heart failure, unspecified | Diagnosis |
|  | 428.1 | Left heart failure | Diagnosis |
|  | 428.20 | Systolic heart failure, unspecified | Diagnosis |
|  | 428.2 | Systolic heart failure | Diagnosis |
|  | 428.21 | Acute systolic heart failure | Diagnosis |
|  | 428.30 | Diastolic heart failure, unspecified | Diagnosis |
|  | 428.3 | Diastolic heart failure | Diagnosis |
|  | 428.31 | Acute diastolic heart failure | Diagnosis |
|  | 428.4 | Combined systolic and diastolic heart failure, unspecified | Diagnosis |
|  | 428.41 | Acute combined systolic and diastolic heart failure | Diagnosis |
|  | 428.9 | Heart failure, unspecified | Diagnosis |
|  | 518.51 | Acute respiratory failure following trauma and surgery | Diagnosis |
|  | 518.81 | Acute respiratory failure | Diagnosis |
|  | 572.2 | Hepatic encephalopathy | Diagnosis |
|  | 584 | Acute renal failure | Diagnosis |
|  | 584.5 | Acute kidney failure with lesion of tubular necrosis | Diagnosis |
|  | 584.6 | Acute kidney failure with lesion of renal cortical necrosis | Diagnosis |
|  | 584.7 | Acute kidney failure with lesion of renal medullary [papillary] necrosis | Diagnosis |
|  | 584.8 | Acute kidney failure with other specified pathological lesion in kidney | Diagnosis |
|  | 584.9 | Acute kidney failure, unspecified | Diagnosis |
|  | 586 | Renal failure, unspecified | Diagnosis |
