# Supplementary material for: Impact of intravenous fluid composition on outcomes in patients with systemic inflammatory response syndrome
Source: Crit Care. 2015 Sep 12;19(1):334. doi: 10.1186/s13054-015-1045-z (PMC4570151; doi:10.1186/s13054-015-1045-z)
Supplement: Additional file 2: Table S2. — Summary of clinical outcomes definitions and criteria. This table summarizes and defines clinical outcomes, grouped by category. (DOCX 18 kb) [file 13054_2015_1045_MOESM2_ESM.docx]

| **Table S2.** Summary of clinical outcomes definitions and criteria | | | | | |
| --- | --- | --- | --- | --- | --- |
| **Clinical Outcome** | | |  |  |  |
|  | | **Timing: Unless otherwise noted, outcomes were analyzed from day of SIRS qualification through patient hospital discharge** | | | |
| **Cardiac Complications** | |  |  |  |  |
|  | | Dysrhythmia | IV infusion of anti-arrhythmics (e.g. amiodarone, diltiazem, esmolol, metoprolol, labetolol, digoxin, etc) on the day of SIRS qualifying event through the following 3 days |  |  |
|  | | Cardiac stress | Abnormal troponin I or T test result flag within database |  |  |
|  | | Heart failure (BNP and diuretics) | Order for ECHO OR BNP test result >600 combined with diuretic ordered same day as test through 3 days |  |  |
|  | | Heart failure (SOFA) | SOFA cardiovascular scores |  |  |
| **Respiratory** | |  |  |  |  |
|  | | Respiratory failure | SOFA pulmonary scores^2^ restricted to patients who had arterial blood gas measurements. Stages 3 & 4 require respiratory support as determined by ventilator procedure codes (from administrative outcome) or presence of values for characteristics indicative of ventilator use such as tidal volume, maximum pressure, maximal (or peak) inspiratory pressure, or ventilator alarm settings |  |  |
| **Gastrointestinal** | |  |  |  |  |
|  | | Hepatic function | SOFA liver scores^2^ |  |  |
| **Clinical Outcome** | | | **Intervention 1** | **Intervention 2** | **Intervention 3** |
|  | *Clostridium difficile* | | Positive *C. diff* toxin, antigen, or culture test | AND white blood cell (WBC) count >12,000/μL +/-1 day of lab/culture order date | AND metronidazole or vancomycin use within 3 days of *C. diff* lab order date |
| **Infection** |  | |  |  |  |
|  | Pneumonia (culture) | | Positive tracheal aspirate or bronchoalveolar (BAL) culture | AND white blood cell (WBC) count >12,000/μL +/-1 day of lab/culture order date | AND antibiotic use within 3 days of culture order date |
|  | Pneumonia (P/F ratio) | | P/F ratio < 300 | AND white blood cell (WBC) count >12,000/μL +/-1 day of lab/culture order date | AND antibiotic use within 3 days of lab order date |
|  | Blood/sepsis | | Positive blood culture | AND white blood cell (WBC) count >12,000/μL +/-1 day of lab/culture order date | AND antibiotic use within 3 days of culture order date |
|  | Urinary tract | | Positive urine culture | AND white blood cell (WBC) count >12,000/μL +/-1 day of lab/culture order date | AND antibiotic use within 3 days of culture order date |
|  | Surgical site | | Positive culture from wound, abscess, or fluid samples from surgical site the day of first resuscitation fluid receipt or after through the following 7 date stamps if any surgical procedures performed during this timeframe | AND white blood cell (WBC) count >12,000/μL +/-1 day of lab/culture order date | AND antibiotic use within 3 days of culture order date |
| **Hemorrhage/**  **Hematologic** |  | |  |  |  |
|  | Bleeding | | > 2 g hemoglobin decrease in 24 hours (for multiple readings on one day, extremes were chosen) |  |  |
|  | Transfusion | | Transfusion as indicated by ICD-9 procedure code 99.0 Transfusion of Blood and Blood Components (also including 99.03 - 99.07 and 99.09) |  |  |
|  | Coagulopathy (excluding patients receiving warfarin) | | Method 1: PT-INR > 1.5 | OR abnormal D-dimer test result per abnormal flag | OR thrombocytopenia  (< 150,000 platelet count) |
|  |  | | Method 2: SOFA coagulation scores^2^ (platelet counts) |  |  |
| **Renal Failure** |  | |  |  |  |
|  | KDIGO stage | | Scores calculated for KDIGO stages^1^ based on serum creatinine levels for 7 days post SIRS qualification. To assess effect of diuretic on renal failure, diuretic use monitored -1 day and +3 days of abnormal serum creatinine reading, and abnormal kidney function assignment only in the absence of diuretic use. |  |  |
| **Electrolyte Abnormalities** |  | |  |  |  |
|  | Magnesium | | Magnesium >2.4 mg/dL or <1.6 mg/dL considered high or low, respectively, and reported separately | Patients with low levels examined for electrolyte replacement with IV or injectable forms of magnesium sulfate |  |
|  | Potassium | | Potassium >5.0 mg/dL or <3.5 mg/dL considered high or low, respectively, and reported separately | Patients with low levels examined for electrolyte replacement with IV or oral forms of potassium chloride or potassium phosphate |  |
|  | Calcium | | Ionized calcium >5.6 mg/dL or <4.5 mg/dL considered high or low, respectively, and reported separately | Patients with low levels examined for electrolyte replacement with IV or injectable forms of calcium chloride or calcium gluconate |  |
|  | Sodium | | Serum sodium >145 mg/dL or <136 mg/dL considered high or low, respectively, and reported separately |  |  |
| **Acidosis** |  | |  |  |  |
|  | Bicarbonate (HCO_3_) | | pH (via arterial blood gas test) <7.35 | AND bicarbonate level < 22 mmol/L |  |
|  | Lactate | | pH (via arterial blood gas test) <7.35 | AND lactate level >2.0 mmol/L from any of the following tests (1) arterial lactate, (2) venous lactate, or (3) blood lactic acid |  |
|  | Hyperchloremia | | pH (via arterial blood gas test) <7.35 | AND chloride test level >100 mmol/L |  |
| ^1^ Kidney Disease: Improving Global Outcomes (KDIGO) Acute Kidney Injury Work Group. KDIGO Clinical Practice Guideline for Acute Kidney Injury. Kidney Int Suppl 2012;2:6–6  ^2^Vincent J.-L., Moreno R., Takala J., Willats S., De Mendonca A., Bruining H., Reinhart C. K., Suter P. M. & Thijs L. G. (1996). The SOFA (Sepsis-related Organ Failure Assessment) score to describe organ dysfunction/failure. On behalf of the Working Group on Sepsis-Related Problems of the European Society of Intensive Care Medicine. *Intensive Care Med,* 22:707-710 | | | | |  |
