# Supplementary material for: Impact of intravenous fluid composition on outcomes in patients with systemic inflammatory response syndrome
Source: Crit Care. 2015 Sep 12;19(1):334. doi: 10.1186/s13054-015-1045-z (PMC4570151; doi:10.1186/s13054-015-1045-z)
Supplement: Additional file 3: Table S3. — SOFA outcomes by treatment group. This table shows the prevalence of SOFA outcomes, categorized by treatment group. (DOCX 12 kb) [file 13054_2015_1045_MOESM3_ESM.docx]

**Table S3.** SOFA Outcomes by Treatment Group

| **Organ System** | **Saline**  **(n = 1558)**  **% (n)** | **Balanced**  **(n = 1558)**  **% (n)** | **p-value** |
| --- | --- | --- | --- |
| **SOFA CV**  0 (Normal)  1  2  3  4 | 92.58 (1447)  6.4 (100)  1.02 (16)  0 (0)  0 (0) | 98.72 (1538)  0.9 (14)  0.39 (6)  0 (0)  0 (0) | <0.0001 |
| **SOFA Pulmonary**  0 (Normal)  1  2  3  4 | 50.63 (40)  10.13 (8)  34.18 (27)  5.06 (4)  0 (0) | 40.54 (30)  10.81 (8)  33.78 (25)  10.81 (8)  4.05 (3) | 0.2242 |
| **SOFA Liver**  0 (Normal)  1  2  3  4 | 89.67 (1397)  7.06 (110)  2.63 (41)  0.45 (7)  0.19 (3) | 94.03 (1465)  3.79 (59)  1.8 (28)  0.39 (6)  0 (0) | 0.0002 |
| **SOFA Hematologic**  0 (Normal)  1  2  3  4 | 56.61 (766)  4.51 (61)  16.78 (227)  19.59 (265)  2.51 (34) | 76.36 (1050)  1.45 (20)  9.24 (127)  11.27 (155)  1.67 (23) | <0.0001 |
| **KDIGO Stage**  Normal  1  2  3 | 94.54 (1473)  3.72 (58)  0.96 (15)  0.77 (12) | 95.57 (1489)  3.27 (51)  0.9 (14)  0.26 (4) | 0.2061 |
